# Can the ketogenic diet improve our dreams? Effect of very low-calorie ketogenic diet (VLCKD) on sleep quality

**DOI:** 10.1186/s12967-023-04280-7

**Published:** 2023-07-18

**Authors:** Luigi Barrea, Ludovica Verde, Cherubino Di Lorenzo, Silvia Savastano, Annamaria Colao, Giovanna Muscogiuri

**Affiliations:** 1 Dipartimento di Scienze Umanistiche, Università Telematica Pegaso, Via Porzio, Centro Direzionale, isola F2, Napoli, 80143 Italy; 2 Unità di Endocrinologia, Diabetologia e Andrologia, Dipartimento di Medicina Clinica e Chirurgia, Centro Italiano per la cura e il Benessere del paziente con Obesità (C.I.B.O), Università degli Studi di Napoli Federico II, Via Sergio Pansini 5, 80131 Naples, Italy; 3 Department of Public Health, University of Naples Federico II, Naples, Italy; 4 Department of Medico-Surgical Sciences and Biotechnologies, Sapienza University of Rome Polo Pontino-ICOT, Latina, 04100 Italy; 5 Unità di Endocrinologia, Diabetologia e Andrologia, Dipartimento di Medicina Clinica e Chirurgia, Università degli Studi di Napoli Federico II, Via Sergio Pansini 5, 80131 Naples, Italy; 6 Cattedra Unesco “Educazione alla salute e allo sviluppo sostenibile”, University Federico II, Naples, Italy

**Keywords:** Sleep quality, Obesity, Very low-calorie ketogenic diet, VLCKD, Ketogenic diet, Fat mass, Sleep management, Nutrition, Diet

## Abstract

**Background:**

Obesity is a condition that is often associated with sleep disorders, including reduced sleep quality (SQ). Very low calorie ketogenic diet (VLCKD) has proven to be effective in the management of obesity and associated metabolic disorders. However, little is still known about the effects of this promising nutritional protocol on SQ. Thus, the purpose of this study was to investigate the short-term effect of VLCKD on SQ in women with overweight/obesity and if any changes, to identify the predictive factor that through VLCKD modified SQ.

**Methods:**

Were consecutively enrolled a total of 324 subjects, who met the inclusion criteria and accepted to adhere to VLCKD. Assessment of nutritional status, including anthropometric measurements (height, weight, and waist circumference), bioelectrical impedance analysis (phase-sensitive system, 50 kHz BIA 101 RJL, Akern Bioresearch, Florence, Italy Akern), high sensitivity C reactive protein levels (hs-CRP), and SQ were carried out at baseline and after 31 days of active stage of VLCKD. SQ was evaluated using the validated questionnaire Pittsburgh Sleep Quality Index (PSQI).

**Results:**

In addition to the expected general improvement of anthropometric parameters and body composition, VLCKD improved significantly SQ, as demonstrated by the improvement of all parameters included in the PSQI questionnaire (p < 0.001). Both at baseline and after 31 days of active stage of VLCKD, the PSQI score was significantly associated with BMI, waist circumference, fat mass, fat free mass (p < 0.001 for all) and hs-CRP (p = 0.023). PhA was negatively associated with PSQI score only at baseline (p < 0.001). ∆% PSQI positively correlated with ∆% BMI, ∆% fat mass, ∆% hs-CRP (p < 0.001 for all) and negatively correlated with ∆% fat free mass (p < 0.001), and ∆% PhA (p = 0.031). In the multiple regression analysis ∆% fat mass represented the only predictor of changes in SQ after VLCKD. Finally, in the ROC analysis, a threshold value of ∆% fat mass > − 8.4% predicted improvement in SQ (p < 0.001).

**Conclusion:**

In conclusion, VLCKD determines an improvement of SQ in women with overweight and obesity, that was mostly mediated by the reduction of fat mass related to this nutritional protocol.

**Graphical Abstract:**

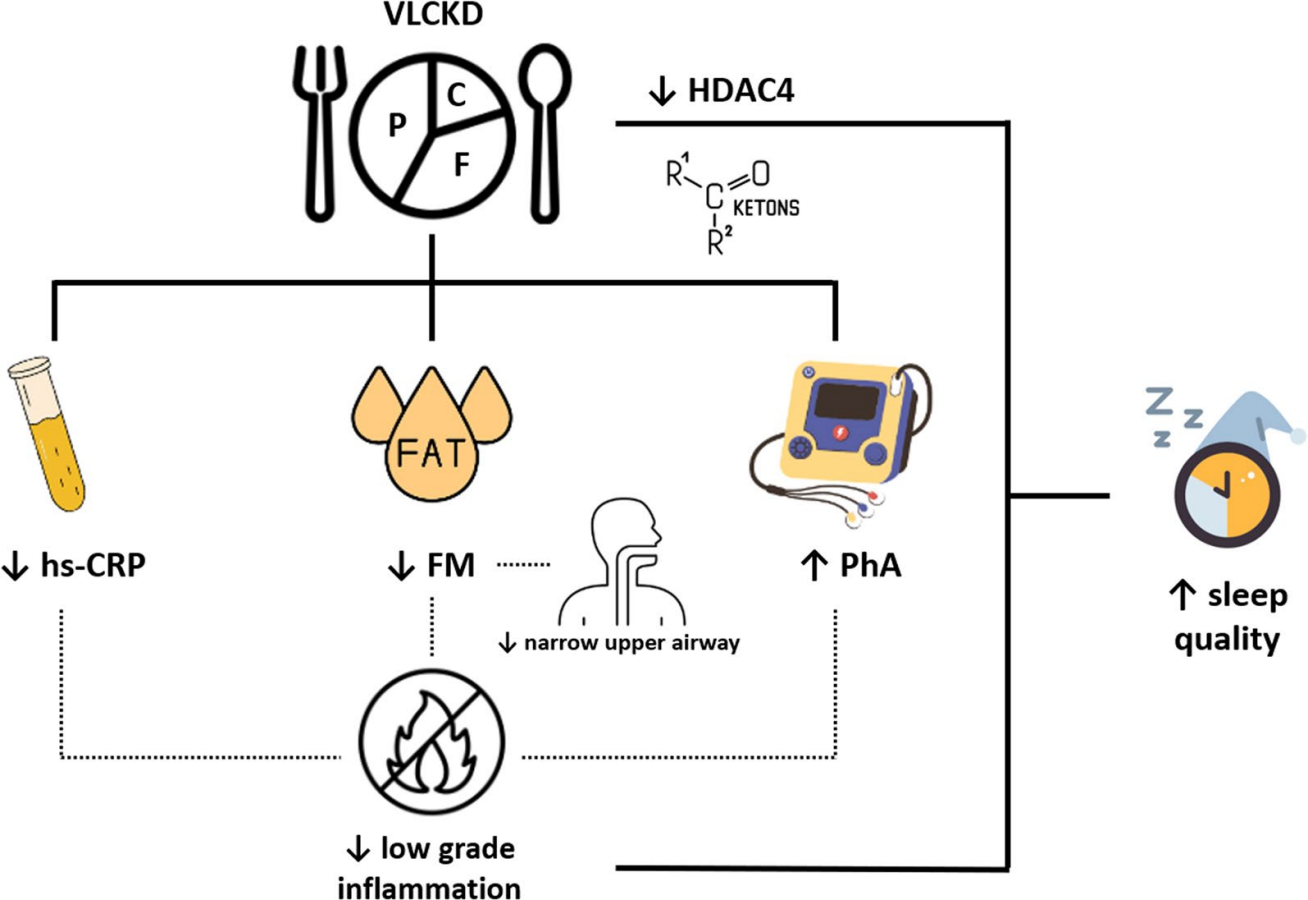

## Introduction

Poor health and an increased risk of chronic non-communicable degenerative diseases [1–3], particularly cardiometabolic diseases [4, 5], have been linked to disruptions in sleep quality. In particular, obesity is a condition that is often associated with sleep disorders, including reduced sleep quality [6]. Nutrition plays a crucial role in determining sleep quality, as evidenced by a study involving 172 middle-aged adults that found good sleepers had higher adherence to the Mediterranean diet than poor sleepers [7]. In this study, consuming the Mediterranean dietary pattern, rather than a single nutrient, was found to have a positive effect on sleep quality [7]. This promising effect of the Mediterranean diet on sleep quality can be attributed to its high levels of polyunsaturated fatty acids and phytochemicals, in particular polyunsaturated fatty acids n-3 and polyphenols, which can reduce inflammation and positively regulate neurotransmission processes, including dopaminergic, serotonergic, cholinergic and glutamatergic systems [8–10]. The Mediterranean diet can also influence melatonin biosynthesis through certain foods and beverages that contain melatonin precursors (namely tryptophan and serotonin) and melatonin itself [11].

Recently, the Very Low-Calorie Ketogenic Diet (VLCKD) has gained popularity as a successful nutritional pattern for managing obesity [12]. VLCKD (total daily energy intake ≃ 800 kcal) involves a significant reduction in daily carbohydrate intake, usually lower than 30 g/day (≃ 13% of total energy intake), with an increase in fat (≃ 44%) and protein intake (≃ 43%), leading to the production of ketone bodies [12].

Of note, VLCKD has been also reported to play some role on sleep [13]. A nutritional intervention study with a follow-up of 4 month enrolled twenty adult subjects with obesity in order to investigate the effect of a VLCKD on lifestyle in terms of food and alcohol cravings, physical, sexual activity and sleep disturbances [13]. Relevantly, at the end of VLCKD the authors detected an improvement of sleepiness [13]. However, little is still known about the effects of this promising nutritional protocol on sleep quality. In this regard, the aim of this study was to investigate the short-term effect of VLCKD on sleep quality in women with overweight/obesity and if any changes, to identify the predictive factors that through VLCKD modified sleep quality. Based on animal evidence [14], we hypothesized that VLCKD could play a role in improving sleep quality in subjects with overweight/obesity.

## Materials and methods

### Design and setting

This was and uncontrolled, single-center, open-label pilot clinical study. Participants were recruited from the Obesity Unit at the University “Federico II” of Naples between May 2021 and November 2022. The study involved two visits: one at the beginning and another after 31 days of following a VLCKD. The study received approval from the Federico II Ethical Committee (file no. 50/20) and was conducted according to the World Medical Association’s Code of Ethics (Declaration of Helsinki) for human studies. All women were informed about the study’s objectives and design and provided their informed consent.

### Population study

To increase the homogeneity of patient samples, we retrospectively focused on data from only Caucasian women with overweight/obesity who wanted to lose weight but had previously failed at dieting. Comprehensive medical information was collected from all participants, and their physical activity levels at baseline were evaluated. Women who performed at least 30 min of aerobic physical activity per day were considered physically active (data were tabulated in dichotomous form “yes” or “no”), as previouslt reported [15, 16]. Individuals eligible for this study satisfied the following criteria: women aged 18–69 years, body mass index (BMI) 25.0–50.9 kg/m^2^. The exclusion criteria were put in place to ensure the study’s homogeneity and included being male, having type 1 or type 2 diabetes mellitus, taking anti-inflammatory drugs within the past month, undergoing prior anti-obesity drug treatment or bariatric surgery, having medical conditions that could affect fluid balance or body composition, such as cancer, liver or kidney failure, menstrual abnormalities, immune and inflammatory diseases, as determined by a medical examination and laboratory tests, and failure to comply with the guidelines or attend follow-up visits. Women with pacemakers or implanted defibrillators and those who had sustained a skin injury where the body impedance analysis (BIA) electrodes were placed were also excluded from the study due to potential counfounding effects on the phase angle (PhA) device. Additionally, reproductive-age participants were assessed during the early follicular phase at both baseline and follow-up.

### Study protocol

**Fig. 1:**
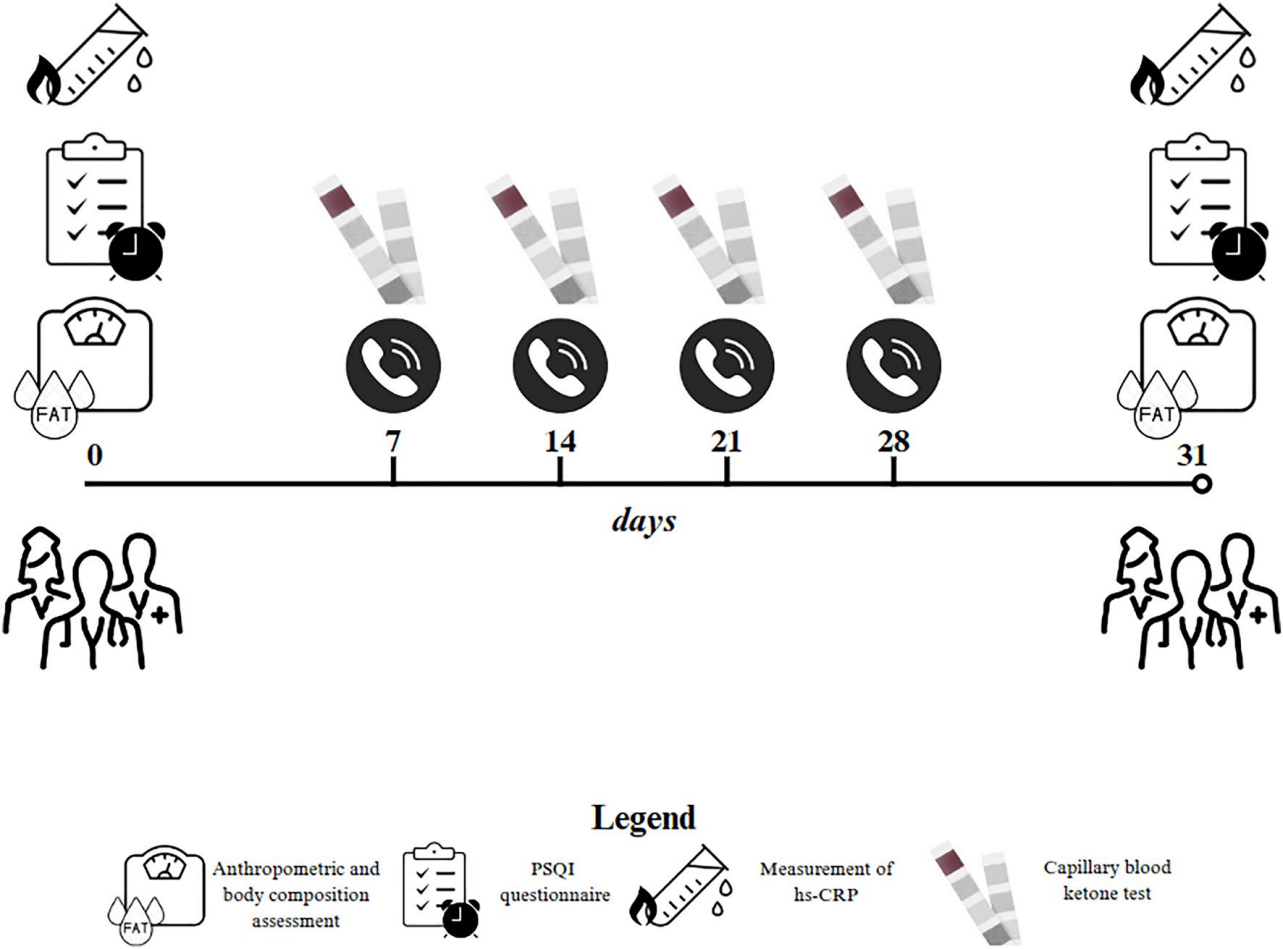
Study protocol

As illustrated in Fig. 1, at the start of the study (T0), all women underwent an assessment by an endocrinologist and a nutritionist to determine if they met the criteria for the study. The endocrinologist obtained their medical history and ruled out any issues that would prevent them from following VLCKD as per the European Association for the Study of Obesity (EASO) guidelines [17]. Afterwards, the nutritionist performed measurements of anthropometric parameters, body composition and sleep quality, and provided clinical nutritional counseling along with instructions for the VLCKD protocol to be followed for a period of 31 days. All women were informed about the VLCKD protocol and the ketur test, and were instructed to maintain their usual physical activity levels throughout the study. The nutritionist contacted each women by phone once a week to check if they were adhering to the VLCKD protocol and to record ketone body measurements from capillary blood. After 31 days, all women underwent a follow-up assessment with the endocrinologist and nutritionist, during which they were evaluated clinically, measured for body composition and inflammation markers, and assessed for sleep quality.

### Anthropometric measurements

A single staff member conducted measurements and evaluations on women in the morning (8:00–10:00 a.m.) after they had fasted overnight for at least 8 h, as previously reported [18–20]. Women wore light clothing and no shoes during the anthropometric measurements. Height and weight measurements were taken using a wall-mounted stadiometer and a calibrated beam scale, respectively, with height measured to the nearest 0.5 cm and weight to the nearest 0.1 kg. The body mass index (BMI) was calculated using these measurements and classified according to the World Health Organization (WHO) criteria as:

- Normal weight (18.5–24.9 kg/m^2^),
- Overweight (25.0–29.9 kg/m^2^),
- Obesity grade I (30.0–34.9 kg/m^2^),
- Obesity grade II (35.0–39.9 kg/m^2^),
- Obesity grade III (40.0 kg/m^2^ or more) [21].

Waist circumference (WC) was measured using a non-stretchable measuring tape to the nearest 0.1 cm at the narrowest point, or at the umbilical level if the narrowest point was not visible, with women standing upright, feet together, arms hanging loosely by their sides, and breathing normally [22].

### Nutritional intervention

Women who met the requirements to participate in the study were put on a VLCKD with the use of replacement meal and which involved three stages: active, re-education, and maintenance [23–25]. Before beginning the active phase of VLCKD, a qualified nutritionist prepared it after evaluating women’s nutritional status, and an endocrinologist prescribed it. The VLCKD was based on a commercial weight-loss program (New Penta, Cuneo, Italy), which restricted total daily energy intake to less than 800 kcal and consisted of 13% carbohydrates (< 30 g/day), 43% protein (1.2–1.5 g/kg ideal body weight), and 44% lipids (mostly from extra-virgin olive oil). The protein sources used in the VLCKD were derived from high-quality preparations made from peas, eggs, soy, and whey. In accordance with international recommendations, women were advised to take multi-vitamin and saline supplements to maintain the body’s acid/base balance (vitamins B, E, C, magnesium, calcium, potassium, sodium, and omega-3 fatty acids).

### Bioelectrical impedance analysis

To ensure consistency and avoid discrepancies in readings due to different devices or observers, a certified clinical nutritionist conducted the test using the same BIA device. The phase-sensitive BIA system (BIA 101 RJL, Akern Bioresearch, Florence, Italy) used was an 800 A current with a frequency of 50 kHz following the guidelines of the ESPEN (European Society of Parenteral and Enteral Nutrition) [26]. Participants were required to lie down with their limbs slightly apart from the body and to abstain from eating, drinking, or exercising for six hours prior to the test, and from drinking alcohol 24 h before the test. The participants removed their shoes and socks before the electrodes were applied, and the contact surfaces were cleaned with alcohol. Electrodes were placed on the right hand and foot, and measurements were taken under standardized conditions with the same device to avoid variations due to observers or devices. The device was checked using resistors and capacitors of known values. The intraday and interday reliability of the measurements by the same observer was < 2% for resistance and < 2.5% for reactance, while the CVs for repeated measurements of resistance and reactance at 50 kHz were 1.4% and 1.3%, respectively, as determined in 10 females by the same observer. The phase angle (PhA) was calculated using the following formula: PhA (°, degrees) = arc tangent reactance/resistance * (180/π) based on the conditions at 50 kHz.

### Adherence to the very low calorie ketogenic diet (VLCKD)

Women’s compliance with the VLCKD and physical activity recommendations was assessed by the endocrinologist and nutritionist through weekly individual telephone counseling sessions using a structured support program. The nutritionist monitored any changes in physical activity levels, which were recorded on a predetermined patient card. Furthermore, once a week, participants performed a capillary blood test at home using test strips (Optium Xceed Blood Glucose and Ketone Monitoring System; Abbott Laboratories, Chicago, IL, USA) to measure their ketosis. The nutritionist provided instructions on how to perform the test before the telephone interview, and the results (either “yes” or “no”) were documented during the interview. All women had a positive blood ketone test.

### Measurement of high sensitivity C reactive protein levels

After an overnight fast of at least 8 h, at baseline and after 31 days of active stage of VLCKD, high sensitivity C reactive protein (hs-CRP) levels were dosed, taken in the morning (8.00–10.00 a.m.), through venous blood samples. The assessment was conducted in a subgroup of 263 women. The hs-CRP levels were evaluated using a high-sensitivity nephelometric assay (CardioPhase hsCRP kit, Siemens Healthcare Diagnostics, Marburg, Germany), with a lower limit of detection of 0.01 mg/L and an intra- and interassay CV of < 7%. Based on the hs-CRP levels measured at baseline and after 31 days of active stage of VLCKD, women were classified into three groups, low cardiovascular risk (< 1.0 mg/L), intermediate cardiovascular risk (1.0–3.0 mg/L), and high cardiovascular risk (≥ 3.0 mg/L), according to ACC/AHA guideline [27].

### Sleep quality

The quality of sleep was evaluated using the validated questionnaire Pittsburgh Sleep Quality Index (PSQI) [28]. This questionnaire consists of seven sessions, including as subjective sleep quality, habitual sleep efficiency, sleep medication use, sleep duration, sleep latency, sleep disturbances, and daytime dysfunction. Each of these seven components is given equal weight and is rated on a scale of zero to three, with three indicating the most negative response on the Likert Scale. The PSQI score is a global score ranging from zero to 21, and poor sleep quality was defined as a PSQI score of 5 or greater, while good sleep quality was defined as a PSQI score of less than 5 [28].

### Power analysis

The power of the sample was calculated by the difference of means ± standard deviation (SD) of the PSQI score pre e post VLCKD (7.74 ± 4.89 vs. 4.97 ± 3.57; respectively). The minimum number of cases required was 162 individuals. The calculated power size was 95%, with a type I (alpha) error of 0.05 (95%), and a type II (beta) of 0.05. The calculations of sample size and power were performed while using a sample size calculator Clinical Calc (https://clincalc.com/stats/samplesize.aspx), as previously reported [29].

### Statistical analysis

Data were analyzed using MedCalc® package (Version 12.3.0 1993–2012 MedCalc Software bvba—MedCalc Software, Mariakerke, Belgium) and IBM SPSS Statistics Software (PASW Version 21.0, SPSS Inc., Chicago, IL, USA). Only women who had both baseline and after 31 days of active stage of VLCKD measurements were included in the statistical analysis. Results were presented as mean ± standard deviation (SD) for continuous variables and as a number and percentage (n, %) for categorical variables. The Kolmogorov–Smirnov test was used to assess data distribution. Skewed variables such as waist circumference (WC), fat mass (FM), and fat free mass (FFM) were normalized using a logarithm and then reconverted into figures and tables. Paired Student’s t-test was used to compare the differences between baseline and after 31 days of active stage of VLCKD measurements; the chi-square (χ^2^) test was used to compare the frequency distribution across BMI, WC, physical activity levels, hs-CRP, and PSQI categories. ANOVA test with Bonferroni test as post-hoc test was used to analyze the differences in PSQI score at baseline and after 31 days of active stage of VLCKD across BMI, WC, physical activity, hs-CRP, and PSQI categories. Spearman’s correlation was used to examine the association between baseline and after 31 days of active stage of VLCKD (percentage changes, delta ∆%). Furthermore, a multiple linear regression analysis model (stepwise method) was used to estimate the predictive value (Δ%) of BMI, resistance, PhA, and hs-CRP levels on the PSQI score as the dependent variable, expressed as R2, beta (β) and t. Receiver operator characteristic (ROC) curve analysis was performed to determine the area under the curve (AUC), criterion, sensitivity, specificity, standard error, and 95% confidence interval (CI), as well as the cut-off value for ∆FM% in detecting improvement in sleep quality (PSQI < 5). To avoid multicollinearity, variables with a variance inflation factor > 10 were excluded.

## Results

Three hundred and twenty-four women with overweight or obesity who met the necessary criteria were included for statistical analysis. Physical activity levels were unchanged in all women, as required at the baseline visit (χ^2^ = 0.01, p = 0.933).

Table 1 reported anthropometric measurements, body composition parameters, and inflammatory biomarker of the study population at baseline and after 31 days of active stage of VLCKD. Weight, BMI, WC, FM, and hs-CRP levels were significantly diminished (p < 0.001). Conversely, PhA and FFM significantly increased (p < 0.001). Therefore, the distribution of women across BMI categories were significantly changed, with an increase in the prevalence of normal-weight women (+ 4.0%, p < 0.001), and a decrease in the prevalence of grade III obesity (− 11.1%, p < 0.001) (Table 2). Similarly, also the number of women with WC < cut-off was increased (+ 13.3%, p < 0.001). Of interest, after 31 days of active stage of VLCKD, the number of participants at high cardiovascular risk decreased (− 28.9%, p < 0.001), with an increased the number of women at low cardiovascular risk (+ 21.6%, p < 0.001).

**Table 1:** Anthropometric characteristics, body composition and inflammatory parameters of the study population at baseline and after 31 days of active stage of VLCKD

Parameters	Baseline Mean ± SD or number (%) N = 324	Day 31 of VLCKD Mean ± SD or number (%) N = 324	∆%	*p-value
Anthropometric parameters				
Weight (kg)	99.93 ± 16.80	87.82 ± 15.62	− 7.3 ± 2.90	**< 0.001**
BMI (kg/m^2^)	35.64 ± 5.51	32.98 ± 5.19		**< 0.001**
WC (cm)	104.83 ± 15.36	98.14 ± 14.34	− 6.22 ± 5.57	**< 0.001**
Body composition				
R (Ω)	476.82 ± 73.75	482.62 ± 69.20	1.75 ± 9.22	**0.013**
Xc (Ω)	47.31 ± 9.98	51.23 ± 9.96	9.85 ± 16.71	**< 0.001**
FFM (kg)	54.37 ± 6.67	53.54 ± 6.54	− 1.45 ± 4.78	**< 0.001**
FM (kg)	40.55 ± 13.36	34.28 ± 12.28	− 15.71 ± 9.99	**< 0.001**
FFM (%)	58.19 ± 7.23	61.93 ± 7.65	6.61 ± 6.23	**< 0.001**
FM (%)	41.81 ± 7.23	38.07 ± 7.65	− 9.06 ± 8.93	**< 0.001**
PhA (°)	5.67 ± 0.89	6.07 ± 0.83	7.91 ± 13.22	**< 0.001**
Inflammatory parameters				
^a^hs-CRP levels (mg/L)	3.45 ± 3.69	1.80 ± 2.32	− 38.42 ± 41.88	**< 0.001**

*SD* standard deviation, *VLCKD* very low-calorie ketogenic diet, *BMI* body mass index, *WC* waist circumference, *BIA* bioelectrical impedance analysis, *R* resistance, *Xc* reactance, *FFM* free fat mass, *FM* fat mass, *PhA* phase angle, *hs-CRP* high-sensitivity C-reactive protein

*A *p* value in bold type denotes a significant difference (*p* < 0.05)

^a^hs-CRP levels were evaluated in a subgroup of 263 women

**Table 2:** Categories of BMI, WC, physical activity, and hs-CRP of the study population at baseline and after 31 days of active stage of VLCKD

Parameters	Baseline Mean ± SD or number (%) n = 324	Day 31 of VLCKD Mean ± SD or number (%) n = 324	∆%	*p-value
BMI categories				
Normal-weight	0, 0%	13, 4.0%	+ 4.0%	χ^2^ = 11.30, **p < 0.001**
Overweight	54, 16.7%	97, 29.9%	+ 13.2%	χ^2^ = 15.23, **p < 0.001**
Grade I obesity	103, 31.8%	105, 32.4%	+ 0.6%	χ^2^ = 0.01, p = 0.933
Grade II obesity	100, 30.8%	78, 24.1%	− 6.8%	χ^2^ = 3.42, p = 0.065
Grade III obesity	67, 20.7%	31, 9.6	− 11.1%	χ^2^ = 14.73, **p < 0.001**
WC categories				
< Cut-off	50, 15.4%	93, 28.7%	+ 13.3%	χ^2^ = 15.83, **p < 0.001**
> Cut-off	274, 84.6%	231, 71.3%	–	
Physical activity				
Yes	102, 31.5%	102, 31.5%	0%	χ^2^ = 0.01, p = 0.933
No	222, 68.5%	222, 68.5%	–	
^a^hs-CRP categories				
< 1.0 mg/L	52, 19.8%	108, 41.1%	+ 21.6%	χ^2^ = 27.17, **p < 0.001**
1.0–3.0 mg/L	96, 36.5%	116, 44.1%	+ 7.6%	χ^2^ = 2.85, p = 0.091
≥ 3.0 mg/L	115, 43.7%	39, 14.8	− 28.9%	χ^2^ = 51.65, **p < 0.001**

*SD* standard deviation, *VLCKD* very low-calorie ketogenic diet, *BMI* body mass index, *WC* waist circumference, *hs-CRP* high-sensitivity C-reactive protein

*A *p* value in bold type denotes a significant difference (p < 0.05)

^a^hs-CRP levels were evaluated in a subgroup of 263 women

**Fig. 2:**
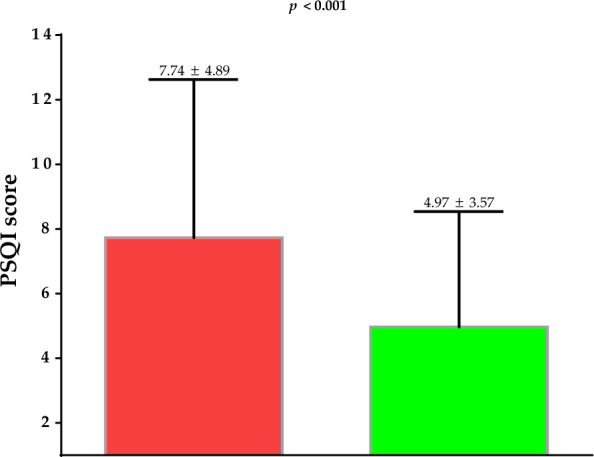
Variation of the PSQI score after 31 days of active stage of VLCKD. *PSQI* Pittsburgh Sleep Quality Index

Table 3 showed the differences of the single items of PSQI questionnaire and PSQI categories of the study population at baseline and after 31 days of active stage of VLCKD. Sleep quality significantly improved after 31 days of active stage of VLCKD as demonstrated by the improvement of all parameters included in PSQI questionnaire (p < 0.001). The 22.2% of participants improved their sleep quality (p < 0.001); Table 3. Figure 2 showed the reduction of the PSQI score after 31 days of active stage of VLCKD (p < 0.001).

**Table 3:** Single items of PSQI and PSQI categories of the study population at baseline and after 31 days of active stage of VLCKD

Parameters of PSQI questionnaire	Baseline Mean ± SD n = 324	Day 31 of VLCKD Mean ± SD n = 324	*p-value
Sleep quality	1.29 ± 0.89	0.92 ± 0.80	**< 0.001**
Sleep onset latency	1.16 ± 1.01	0.82 ± 0.91	**< 0.001**
Sleep duration	1.24 ± 1.06	0.77 ± 0.91	**< 0.001**
Sleep efficiency	0.99 ± 1.08	0.51 ± 0.79	**< 0.001**
Sleep disturbance	1.37 ± 0.84	1.05 ± 0.65	**< 0.001**
Hypnotic drugs	0.51 ± 1.02	0.18 ± 0.62	**< 0.001**
Daytime dysfunction	1.17 ± 1.03	0.72 ± 0.80	**< 0.001**
PSQI categories			
Good sleepers (n, %)	139, 42.9%	211, 65.1%	χ^2^ = 31.32
Poor sleepers (n, %)	185, 57.1%	113, 34.9%	**p < 0.001**

*PSQI* Pittsburgh sleep quality index, *SD* standard deviation, *VLCKD* very low-calorie ketogenic diet

*A *p* value in bold type denotes a significant difference (p < 0.05)

Table 4 reported age, anthropometric measurements, physical activity levels, body composition parameters, and inflammatory biomarker of the study population baseline grouped according to the PSQI categories at baseline and after 31 days of active stage of VLCKD. As reported in the table, stratifying the sample population according to the good and poor sleepers both at baseline and after 31 days of active stage of VLCKD, poor sleepers had higher BMI (p < 0.001), WC (p < 0.001), did less physical activity, had worst body composition parameters (p < 0.001), with higher FM (p < 0.001) and lower FFM (p < 0.001), and had higher hs-CRP levels than good sleepers.

**Table 4:** Age, anthropometric measurements, physical activity levels, body composition parameters, and inflammatory biomarker of the study population baseline grouped according to the PSQI categories

Parameters	Participants n = 324					
	Baseline			Day 31 of VLCKD		
	Good sleepers Mean ± SD or number (%) n = 139	Poor sleepers Mean ± SD or number (%) n = 185	*p-value	Good sleepers Mean ± SD or number (%) n = 211	Poor sleepers Mean ± SD or number (%) n = 113	*p-value
Age (years)	39.32 ± 15.07	38.14 ± 13.91	0.468	38.61 ± 14.63	38.71 ± 14.05	0.954
Weight (kg)	84.12 ± 10.94	103.06 ± 15.84	**< 0.001**	81.94 ± 11.27	98.80 ± 16.68	**< 0.001**
BMI (kg/m^2^)	32.25 ± 4.24	38.19 ± 4.96	**< 0.001**	31.04 ± 4.17	36.59 ± 4.98	**< 0.001**
Normal-weight	0, 0%	0, 0%	–	13, 6.2%	0, 0%	χ^2^ = 5.74, **p = 0.017**
Overweight	46, 33.1%	8, 4.3%	χ^2^ = 45.25, **p < 0.001**	83, 39.3%	14, 12.4%	χ^2^ = 24.21, **p < 0.001**
Grade I obesity	59, 42.4%	44, 23.8%	χ^2^ = 11.90, **p = 0.001**	76, 36.0%	29, 25.7%	χ^2^ = 3.15, p = 0.076
Grade II obesity	28, 20.1%	72, 38.9%	χ^2^ = 12.25, **p = 0.001**	34, 16.1%	44, 38.9%	χ^2^ = 19.74, **p < 0.001**
Grade III obesity	6, 4.3%	61, 33.0%	χ^2^ = 38.01, **p < 0.001**	5, 2.4%	26, 23.0%	χ^2^ = 33.88, **p < 0.001**
WC (cm)	97.97 ± 12.01	109.98 ± 15.60	**< 0.001**	94.17 ± 12.21	105.56 ± 15.13	**< 0.001**
< Cut-off	38, 27.3%	12, 6.5%	χ^2^ = 24.87, **p < 0.001**	77, 36.5%	16, 14.2%	χ^2^ = 16.86, **p < 0.001**
> Cut-off	101, 72.7%	173, 93.5%		134, 63.5%	97, 85.8%	
Physical activity						
Yes	58, 41.7%	44, 23.8%	χ^2^ = 11.03, **p = 0.001**	79, 37.4%	23, 20.4%	χ^2^ = 9.18, **p = 0.002**
No	81, 58.3%	141, 76.2%		132, 62.6%	90, 79.6%	
BIA parameters						
R (Ω)	479.40 ± 66.13	474.89 ± 79.12	0.586	480.90 ± 68.97	485.85 ± 69.84	0.540
Xc (Ω)	50.14 ± 9.66	45.17 ± 9.70	**< 0.001**	51.43 ± 9.67	50.85 ± 10.53	0.620
FFM (kg)	52.57 ± 5.54	55.78 ± 7.17	**< 0.001**	52.60 ± 6.06	55.31 ± 7.04	**< 0.001**
FM (kg)	31.54 ± 7.65	47.34 ± 12.72	**< 0.001**	29.31 ± 8.20	43.58 ± 13.22	**< 0.001**
FFM (%)	62.88 ± 5.16	54.68 ± 6.54	**< 0.001**	64.72 ± 6.34	56.73 ± 7.17	**< 0.001**
FM (%)	37.11 ± 5.16	45.32 ± 6.54	**< 0.001**	35.28 ± 6.34	43.27 ± 7.17	**< 0.001**
PhA (°)	5.98 ± 0.82	5.45 ± 0.87	**< 0.001**	6.12 ± 0.82	5.98 ± 0.85	0.154

	n = 114	n = 149		n = 177	n = 86	
^a^hs-CRP levels (mg/L)	2.64 ± 3.45	3.78 ± 3.61	**0.007**	1.57 ± 2.05	2.27 ± 2.74	**0.021**
< 1.0 mg/L	31, 27.2%	21, 14.1%	χ^2^ = 6.19, **p = 0.013**	80, 45.2%	28, 32.6%	χ^2^ = 5.14, **p = 0.023**
1.0–3.0 mg/L	49, 43.0%	47, 31.5%	χ^2^ = 3.17, p = 0.075	77, 43.5%	39, 45.3%	χ^2^ = 1.96, p = 0.161
≥ 3.0 mg/L	34, 29.8%	81, 54.4%	χ^2^ = 14.82, **p < 0.001**	20, 11.3%	19, 22.1%	χ^2^ = 1.36, p = 0.244

*PSQI* Pittsburgh Sleep Quality Index, *VLCKD* very low-calorie ketogenic diet, *SD* standard deviation, *BMI* body mass index, *WC* waist circumference, *R* resistance, *Xc* reactance, *FFM* free fat mass, *FM* fat mass, *PhA* phase angle, *hs-CRP* high-sensitivity C-reactive protein

*A p value in bold type denotes a significant difference (p < 0.05).

^a^hs-CRP levels were evaluated in a subgroup of 263 women

**Fig. 3:**
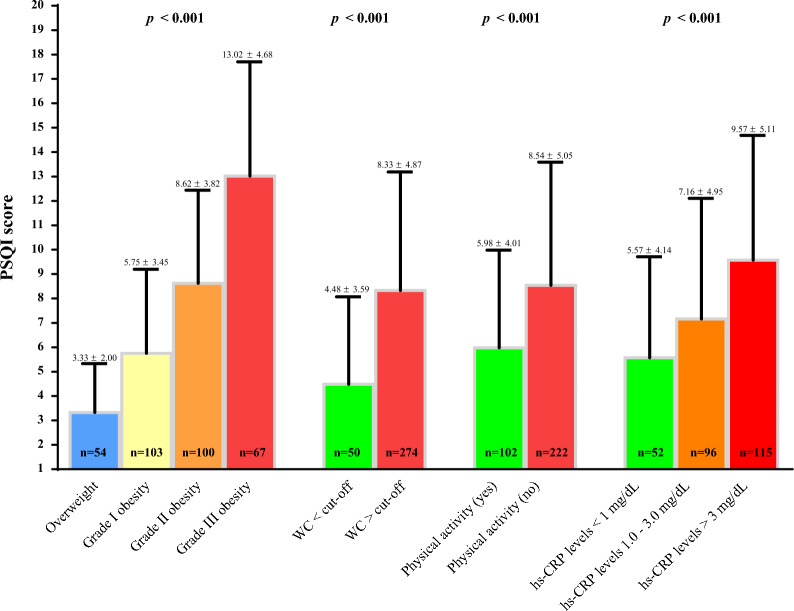
PSQI score in the different categories of BMI, WC, and physical activity at baseline. A p value in bold type denotes a significant difference (p < 0.05). hs-CRP levels were evaluated in a subgroup of 263 women. *PSQI* Pittsburgh Sleep Quality Index, *BMI* body mass index, *WC* waist circumference, *hs-CRP* high-sensitivity C-reactive protein

**Fig. 4:**
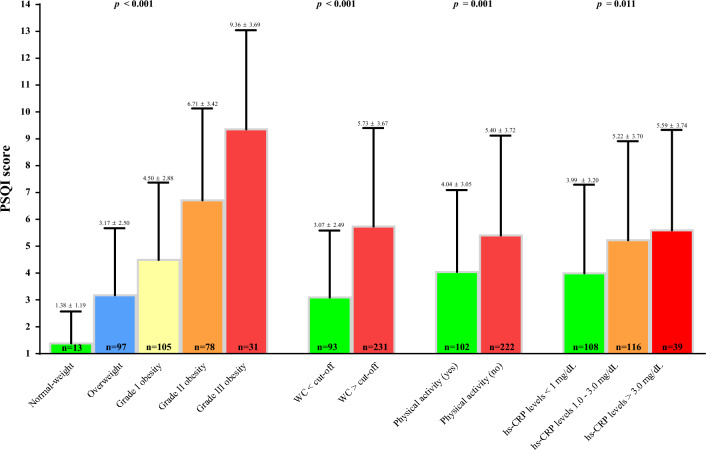
PSQI score in the different categories of BMI, WC, and physical activity after 31 days of active stage of VLCKD. A p value in bold type denotes a significant difference (p < 0.05). hs-CRP levels were evaluated in a subgroup of 263 women. *PSQI* Pittsburgh Sleep Quality Index, *BMI* body mass index, *WC* waist circumference, *hs-CRP* high-sensitivity C-reactive protein

Figures 3 and 4 showed PSQI score in the different categories of BMI, WC, physical activity, and hs-CRP levels at baseline and after 31 days of active stage of VLCKD, respectively. Both at baseline and after 31 days of active stage of VLCKD, the PSQI score was higher in women with grade III obesity (p < 0.001), WC > cutoffs (p < 0.001), who did not exercise, and with hs-CRP levels greater than 3 mg/dL; Figs. 3 and 4, respectively.

Correlations of PSQI score with age, anthropometric measurements, body composition parameters, and inflammatory biomarker of the study at baseline and after 31 days of active stage of VLCKD were reported in Table 5. Both at baseline and after 31 days of active stage of VLCKD, the PSQI score was significantly associated with weight (p < 0.001), BMI (p < 0.001), WC (p < 0.001), FM (p < 0.001), FFM (p < 0.001), and hs-CRP levels (p = 0.023). PhA was negatively associated with PSQI score only at baseline (p < 0.001).

**Table 5:** Correlation between PSQI score with age, anthropometric measurements, body composition parameters, and inflammatory biomarker of the study at baseline and after 31 days of active stage of VLCKD

n = 324	Baseline		Day 31 of VLCKD	
Parameters	r	*p-value	r	*p-value
Age (years)	− 0.039	0.487	− 0.004	0.945
Weight (kg)	0.756	**< 0.001**	0.641	**< 0.001**
BMI (kg/m^2^)	0.677	**< 0.001**	0.615	**< 0.001**
WC (cm)	0.541	**< 0.001**	0.498	**< 0.001**
BIA parameters				
R (Ω)	0.013	0.821	0.071	0.200
Xc (Ω)	− 0.262	**< 0.001**	0.001	0.996
FFM (kg)	0.282	**< 0.001**	0.229	**< 0.001**
FM (kg)	0.808	**< 0.001**	0.699	**< 0.001**
FFM (%)	− 0.734	**< 0.001**	− 0.631	**< 0.001**
FM (%)	0.734	**< 0.001**	0.631	**< 0.001**
PhA (°)	− 0.355	**< 0.001**	− 0.079	0.154
^a^hs-CRP levels (mg/L)	0.269	**< 0.001**	0.141	**0.023**

*PSQI* Pittsburgh Sleep Quality Index, *SD* standard deviation, *VLCKD* very low-calorie ketogenic diet, *BMI* body mass index, *WC* waist circumference, *R* resistance, *Xc* reactance, *FFM* free fat mass, *FM* fat mass, *PhA* phase angle, *hs-CRP* high-sensitivity C-reactive protein

*A p value in bold type denotes a significant difference (p < 0.05).

^a^hs-CRP levels were evaluated in a subgroup of 263 women

Table 6 summarized the correlation between ∆% PSQI with age, ∆% anthropometric measurements, ∆% body composition parameters, and ∆% of inflammatory biomarker. As showed, ∆% PSQI positively correlated with ∆% BMI (p < 0.001), ∆% R (p < 0.001), ∆% FM (p < 0.001), ∆% hs-CRP levels (p < 0.001) and negatively correlated with ∆% FFM (p < 0.001), and ∆% PhA (p = 0.031).

**Table 6:** Correlation between ∆% PSQI with age, ∆% anthropometric parameters, ∆% body composition parameters, and ∆% of inflammatory biomarker

Parameters	r	*p*-value
Age (years)	0.007	0.897
∆ BMI (%)	0.234	< 0.001*
∆ WC (%)	0.094	0.090
∆ R (%)	0.319	**< 0.001**
∆ Xc (%)	0.104	0.062
∆ FFM (%)	− 0.376	**< 0.001**
∆ FM (%)	0.370	**< 0.001**
∆ PhA (%)	− 0.120	**0.031**
^a^∆ hs-CRP levels (%)	0.230	**< 0.001**

*PSQI* Pittsburgh Sleep Quality Index, *SD* standard deviation, *VLCKD* very low-calorie ketogenic diet, *BMI* body mass index, *WC* waist circumference, *R* resistance, *Xc* reactance, *FFM* free fat mass, *FM* fat mass, *PhA* phase angle, *hs-CRP* high-sensitivity C-reactive protein

*A p value in bold type denotes a significant difference (p < 0.05)

^a^hs-CRP levels were evaluated in a subgroup of 263 women

To compare the relative predictive power of Δ% BMI, Δ% resistance, Δ% PhA, and Δ% hs-CRP levels associated with ∆% PSQI score, we performed a multiple regression analysis (Model 1) showed in Table 7. In this model, ∆% FM was entered at the first step (p < 0.001) and represents the only predictor of changes in sleep quality after 31 days of active stage of VLCKD.

**Table 7:** Multiple regression analysis models (stepwise method) with ΔPSQI score as a dependent variable to estimate the predictive value of changes in BMI, body composition parameters, and inflammatory biomarker after 31 days of active stage of VLCKD

Parameters	Multiple regression analysis			
	R^2^	*β*	*t*	*p value
Δ% FM kg	0.201	− 0.452	− 8.19	**< 0.001**
Variables excluded: Δ% BMI, Δ% R, Δ% PhA, ^a^Δ% hs-CRP levels				

*PSQI* Pittsburgh Sleep Quality Index, *BMI* body mass index, *VLCKD* very low-calorie ketogenic diet, *FM* fat mass, *R* resistance, *PhA* phase angle, *hs-CRP* high-sensitivity C-reactive protein

*A p value in bold type denotes a significant difference (p < 0.05)

^a^hs-CRP levels were evaluated in a subgroup of 263 women

**Fig. 5:**
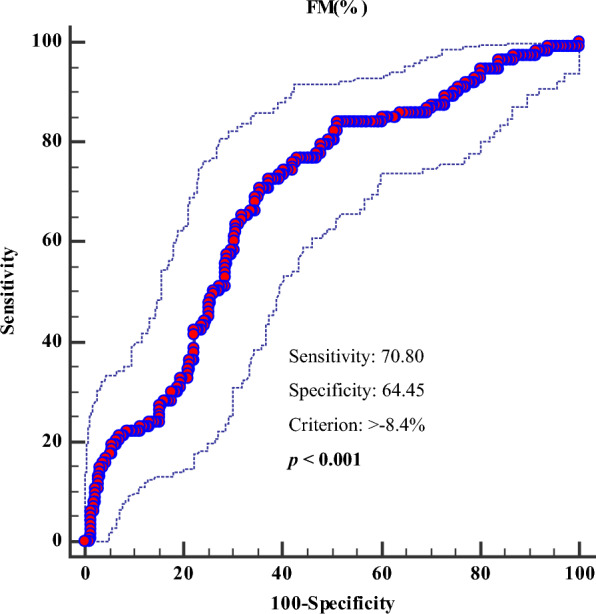
Cut-off value of ∆FM (%) predictive of improvement in sleep quality (PSQI < 5). *FM* fat mass, *PSQI* Pittsburgh Sleep Quality Index

Finally, to determine the cut-off value of ∆% FM that was predictive of improvement in sleep quality (PSQI < 5) a ROC analysis was performed. In the ROC analysis, the threshold value of ∆% FM > − 8.4% predicted improvement in sleep quality (p < 0.001, AUC 0.686, standard error 0.0305, 95% CI = 0.626 to 0.746; Fig. 5).

## Discussion

This study demonstrated that VLCKD induced a severe body weight reduction concomitantly with a decrease in FM, WC, FFM, low grade inflammation measured by hs-CRP levels and PhA and improvements in sleep quality. As previously reported, VLCKD has been demonstrated an effective nutritional protocol able to significantly reduce anthropometric parameters in a short-term period [12, 17, 30–32]. The reduction in anthropometric parameters was also accompanied by an improvement of low-grade inflammation as demonstrated by the decrease of hs-CRP levels and increase of PhA. This latter agreed with a previous study carried out in subjects with obesity undergoing to VLCKD and experiencing an improvement of low-grade inflammation [30]. This anti-inflammatory effect is due not only to VLCKD-related weight loss but also to ketone bodies produced during a ketogenic diet that are able to exert fast effective anti-inflammatory effects through several mechanisms, as the activation of the receptor HCA (hydroxy-carboxylic acid receptor)-2 and PPAR-γ (peroxisome proliferator-activated receptor gamma), inhibition of NF-κB (nuclear factor kappa B) activation, inhibition of NLRP3 inflammasome [33]. Further, the reduction of carbohydrate consumption in the VLCKD lowering chronic inflammation and the risk of obesity-related diseases leads to fatty acid mobilization by adipose tissue that in turn are oxidized to produce ketone bodies, an important energy substrate, leading to reduced body weight and subsequently the inflammatory processes [34]. In this *scenario*, we previously highlighted that VLCKD could become a promising therapeutical tool in psoriasis, a chronic skin immune-mediated disease, characterized by chronic underlying inflammation [35]. As well-known, low-grade inflammation plays a pivotal role in sleep disturbances in obesity [6]. Indeed, we found that subjects with poor sleep quality had significantly higher levels of hs-CRP levels and lower levels of PhA, along with higher values of BMI, WC, FM and FFM compared to subjects with good sleep quality and experienced an improvement of all these parameters after VLCKD. In addition, subjects with good sleep quality were more physically active than subjects with poor sleep quality. Interestingly PSQI score was significantly associated with weight, BMI, WC, FM, FFM and hs-CRP levels at the baseline and at the end of the study while PhA was negatively associated with PSQI score only at baseline. Among these factors the most determinant factor that oversaw improvement of sleep quality was the reduction of FM. As well known, FM and in particular abdominal fat is the main sources of secretion of pro-inflammatory cytokines, such as IL-1, IL-6 and TNF-α [36]. Several evidence reported that pro-inflammatory cytokines could be involved in sleep regulation and thus named as “sleep-regulatory substances” [37, 38]. IL-1β and TNF-α play a role in the physiological regulation of sleep in both animals and humans, and whose secretion follows a circadian rhythm, with the highest IL-6 and TNF-α secretion during the night (between 01:00 and 02:00 h) [39], in particular being involved in the slow-wave sleep (SWS) [40]. In addition, subjects with obesity have a narrow upper airway, typically determined by fat deposition in the parapharyngeal fat pads and pharyngeal muscles that could be responsible of sleep disturbances [41].The reduction of FM experienced in the studied subjects could also contribute to enlarge upper airway thus improving sleep quality. In addition, ketone bodies and in particular β-hydroxybutyrate (β-OHB) has been reported to inhibit histone deacetylases (HDACs) 1, 3, and 4 in human embryonic kidney cells [42]. HDAC 4 participate to a signaling cascade that regulates sleep and wakefulness and the reduction of its activity has been associated to increased sleep [43].

Our study has several strengths but also limitations. First, this pilot study of a large adult population has been carried out in a single center that may have introduced some selection bias. Nevertheless, considering the clear gender differences in sleep quality, we try to reach population homogeneity enrolling only women in reproductive age that were assessed at the same menstrual phase. As a pilot study, we limit our observations to the end of the active stage, and we did not consider the subsequent VLCKD stages. This is because the very short observation period avoid patient drop out. In addition, Although we cannot exclude if the improvement of sleep quality was due to VLCKD per se o to weight loss or to both of them, our study highlights that VLCKD improved sleep quality, and this excludes that this nutritional pattern could have a null or even harmful effect on these parameters. A control design study will need to identify if VLCKD improves sleep quality just through weight loss, as it could occur with other nutritional patterns, and/or through additional mechanisms. Even if sleep quality was assessed by a questionnaire, the strengths of using this tool were: (1) it was a validated questionnaire already used in several studies; (2) it was administered by the same nutritionist both at the baseline and follow up in order to reduce any bias. Finally, although we had no quantitative data on adherence to VLCKD, the nutritionist provided weekly telephone counselling and guided the participants in performing capillary blood tests to measure ketosis. All women had positive blood ketone test results, suggesting compliance with diet recommendations.

## Conclusions

In conclusion, VLCKD determined an improvement of sleep quality in women with overweight and obesity, that was mostly mediated by the reduction of FM related to this nutritional protocol.

Overall, this study highlights the potential benefits of VLCKD not only in managing obesity and metabolic disorders but also in improving sleep quality in women with overweight or obesity. It underscores the importance of considering sleep quality as a relevant outcome and a potential target for intervention in the context of obesity management. However, further studies are needed to confirm these promising results that potentially could lead to make VLCKD a suitable nutritional approach in subjects with obesity and sleep disturbances.

## Data Availability

The datasets used and/or analysed during the current study are available from the corresponding author on reasonable request.

## Abbreviations

VLCKD: Very low-calorie ketogenic diet
WHO: World Health Organization
WC: Waist circumference
BIA: Biompedance analysis
CV: Coefficient of variation
R: Resistance
Xc: Reactance
PhA: Phase angle
FM: Fat mass
FFM: Fat free mass
PSQI: Pittsburgh sleep quality index
hs-CRP: High sensitivity C reactive protein
AUC: Area under the curve
CI: Confidence interval
ROC: Receiver Operating Characteristic
IL: Intereleukin
TNFα: Tumor necrosis factor α
SWS: Slow wave sleep
β-OHB: β-Hydroxybutyrate
HDAC: Histone deactylase
